# Lung and Pleural Findings of Children with Pulmonary Vein Stenosis with and without Aspiration: MDCT Evaluation

**DOI:** 10.3390/children9040543

**Published:** 2022-04-12

**Authors:** Abbey J. Winant, Ryan Callahan, Sara O. Vargas, Kathy J. Jenkins, Vanessa Rameh, Patrick R. Johnston, Maria Niccum, Mirjam L. Keochakian, Edward Y. Lee

**Affiliations:** 1 Department of Radiology, Boston Children’s Hospital, Harvard Medical School, 300 Longwood Avenue, Boston, MA 02115, USA; abbey.winant@childrens.harvard.edu (A.J.W.); vanessa.rameh@childrens.harvard.edu (V.R.); patrick.johnston@childrens.harvard.edu (P.R.J.); 2 Department of Cardiology, Boston Children’s Hospital, Harvard Medical School, 300 Longwood Avenue, Boston, MA 02115, USA; Ryan.Callahan@cardio.chboston.org (R.C.); kathy.jenkins@childrens.harvard.edu (K.J.J.); mirjam.keochakian@childrens.harvard.edu (M.L.K.); 3 Department of Pathology, Boston Children’s Hospital, Harvard Medical School, 300 Longwood Avenue, Boston, MA 02115, USA; sara.vargas@childrens.harvard.edu; 4 Department of Cardiology, Children’s Hospital of Philadelphia, 3401 Civic Center Blvd, Philadelphia, PA 19104, USA; niccumm@chop.edu

**Keywords:** pulmonary vein stenosis, aspiration, multidetector computed tomography (MDCT), lung and pleural abnormalities, children, pediatric patients

## Abstract

Purpose: To retrospectively compare the lung and pleural findings in children with pulmonary vein stenosis (PVS) with and without aspiration on multidetector computed tomography (MDCT). Materials and Methods: All consecutive children (≤18 years old) with PVS who underwent thoracic MDCT studies from August 2004 to December 2021 were categorized into two groups: children with PVS with aspiration (Group 1) and children with PVS without aspiration (Group 2). Two independent pediatric radiologists retrospectively evaluated thoracic MDCT studies for the presence of lung and pleural abnormalities as follows: (1) in the lung (ground-glass opacity (GGO), consolidation, nodule, mass, cyst(s), interlobular septal thickening, and fibrosis) and (2) in the pleura (thickening, effusion, and pneumothorax). Interobserver agreement between the two reviewers was evaluated by the proportion of agreement and the Kappa statistic. Results: The final study population consisted of 64 pediatric patients (36 males (56.3%) and 43 females (43.7%); mean age, 1.7 years; range, 1 day–17 years). Among these 64 patients, 19 patients (29.7%) comprised Group 1 and the remaining 45 patients (70.3%) comprised Group 2. In Group 1 (children with PVS with aspiration), the detected lung and pleural MDCT abnormalities were: GGO (17/19; 89.5%), pleural thickening (17/19; 89.5%), consolidation (16/19; 84.5%), and septal thickening (16/19; 84.5%). The lung and pleural MDCT abnormalities observed in Group 2 (children with PVS without aspiration) were: GGO (37/45; 82.2%), pleural thickening (37/45; 82.2%), septal thickening (36/45; 80%), consolidation (3/45; 6.7%), pleural effusion (1/45; 2.2%), pneumothorax (1/45; 2.2%), and cyst(s) (1/45; 2.2%). Consolidation was significantly more common in pediatric patients with both PVS and aspiration (Group 1) (*p* < 0.001). There was high interobserver agreement between the two independent reviewers for detecting lung and pleural abnormalities on thoracic MDCT studies (Kappa = 0.98; CI = 0.958, 0.992). Conclusion: Aspiration is common in pediatric patients with PVS who undergo MDCT and was present in nearly 30% of all children with PVS during our study period. Consolidation is not a typical radiologic finding of PVS in children without clinical evidence of aspiration. When consolidation is present on thoracic MDCT studies in pediatric patients with PVS, the additional diagnosis of concomitant aspiration should be considered.

## 1. Introduction

In the past decade, substantial advances have been made in elucidating the underlying pathophysiology, diagnosis, and management of pediatric pulmonary vein stenosis (PVS), which is characterized by a decreased luminal caliber of one or more large (extra-pulmonary) pulmonary veins [1,2,3,4,5,6,7,8]. In addition, several studies have evaluated risk factors for poor outcomes in pediatric patients with PVS. These studies found that poor outcomes from PVS in children are associated with bilateral pulmonary vein involvement, smaller size and younger age at diagnosis, and underlying concomitant lung disease [9,10,11,12,13,14,15,16]. Among the studies investigating risk factors for poor outcomes in children with PVS, the most recent study, which demonstrated an association between aspiration and poor outcome in pediatric PVS, is receiving special attention because aspiration is a potentially modifiable risk factor [17].

Among the currently available studies on diagnosing PVS in children, multidetector computed tomography (MDCT), which can non-invasively detect both the decreased caliber of affected pulmonary veins and secondary pleuropulmonary abnormalities in pediatric patients with PVS, has become the imaging modality of choice for detecting PVS in infants and children [18,19,20,21,22,23]. Although early and accurate detection of aspiration-related lung changes has great potential to improve outcomes in children with PVS (because aspiration is a potentially modifiable risk factor), to our knowledge, there is no study which has specifically investigated lung and pleural abnormalities in pediatric patients with PVS and aspiration. Therefore, the purpose of this study was to retrospectively compare the lung and pleural findings in children with PVS with and without aspiration on MDCT.

## 2. Materials and Methods

### 2.1. Institutional Review Board Approval

The Boston Children’s Hospital Institutional Review Board approved this retrospective study which was performed in accordance with the Declaration of Helsinki.

### 2.2. Patient Population

Our hospital’s computerized database from the cardiology department was used to identify all consecutive pediatric patients (≤18 years old) with PVS. The resultant patient list was reviewed and categorized into two groups: Group 1 (PVS and aspiration) and Group 2 (PVS without aspiration). Subsequently, our hospital’s computerized database from the radiology department was used to identify patients with available thoracic MDCT studies in Group 1 and Group 2. For each patient, only the initial thoracic MDCT study performed at the time of diagnosis was included. Using our hospital’s computerized database from the pathology department, available pathology information from patients included in Group 1 and Group 2 was evaluated. The patients’ demographic information was also collected and recorded.

### 2.3. Pulmonary Vein Stenosis and Aspiration Diagnostic Criteria

Diagnostic criteria used for diagnosing for PVS was pulmonary vein luminal narrowing in ≥2 vessels with a mean gradient ≥4 mm Hg seen on echocardiography or conventional angiography [9,18]. The presence of aspiration was documented by referring physicians and/or medical specialists.

### 2.4. Thoracic MDCT Technical Factors

#### 2.4.1. Types of MDCT Scanners

For Group 1, four different MDCT scanners were used for 19 thoracic MDCT studies in the final study group, including: (1) a 16-MDCT scanner (*n* = 1; 5.3%); (2) a 64-MDCT scanner (*n* = 5; 26.3%); (3) a 96-MDCT scanner (*n* = 12; 63.1%); and (4) a 128-MDCT scanner (*n* = 1; 5.3%).

For Group 2, four different MDCT scanners were used for 45 thoracic MDCT studies in the final study group, including: (1) a 16-MDCT scanner (*n* = 5; 11.1%); (2) a 64-MDCT scanner (*n* = 21; 46.7%); (3) a 96-MDCT scanner (*n* = 18; 40.0%); and (4) a 320-MDCT scanner (*n* = 1; 2.2%).

#### 2.4.2. Thoracic MDCT Technical Parameters

All thoracic MDCT studies included in the study were obtained using low radiation dose MDCT techniques closely following the ALARA (As Low As Reasonably Achievable) principle. Technical thoracic MDCT parameters used were weight-based kilovoltage, tube current with radiation dose modulation techniques, and fast tube rotation time (≤1 s) with anatomic coverage from the thoracic inlet to the level of diaphragm. All thoracic MDCT studies included in both Group 1 and Group 2 were obtained with intravenous contrast (1.5–2.0 mL/kg).

### 2.5. Thoracic MDCT Image Review

After obtaining the axial CT data set, two-dimensional reformatted MDCT images were reconstructed using the thin section CT images (≤1 mm) in both coronal and sagittal planes. The reviewers evaluated these axial and two-dimensional reformatted MDCT images on a picture archiving and communication system (PACS) (Synapse, Fujifilm Medical System, Stamford, CT, USA) after removing patient identifiers and randomizing all thoracic MDCT images in both Group 1 and Group 2.

Subsequently, two independent pediatric radiologists, who were blinded to patients’ clinical information and the original report findings of the thoracic MDCT studies, independently reviewed all thoracic MDCT studies. These two independent reviewers were a pediatric thoracic radiologist and a pediatric radiology fellow with 11 and 5 years of experience in interpreting pediatric thoracic MDCT studies, respectively. When there were disagreements between two initial reviewers, a third pediatric radiologist, with more than 20 years of experience in interpreting thoracic MDCT studies, served as a tie breaker and rendered the final decision.

### 2.6. Thoracic MDCT Study Image Assessment

The reviewers systematically evaluated all thoracic MDCT studies for the presence of lung and pleural abnormalities based on previously established criteria [24,25].

#### 2.6.1. Lung Evaluation on Thoracic MDCT Study

The reviewers evaluated the lungs on the included thoracic MDCT studies for the presence of seven main potential abnormalities: (1) ground-glass opacity (GGO); (2) consolidation; (3) nodule; (4) mass; (5) cyst(s); (6) septal thickening; and (7) fibrosis [24]. The diagnosis of GGO was made when there was an area of hazy, increased lung opacity with indistinct margins of pulmonary vessels. When there was a homogeneous increase in pulmonary parenchyma attenuation which obscured the margins of adjacent vessels and airway walls, the diagnosis of consolidation was made. Of note, atelectasis instead of consolidation was considered to be present when there was a reduced lung volume accompanied by increased pulmonary parenchymal attenuation. A nodule was deemed present when a rounded or irregular opacity measuring up to 3 cm in diameter was seen. The diagnosis of a mass was made when there was a solid or partly solid opacity that was larger than 3 cm in diameter. When a round parenchymal lucency or a low-attenuating area with a well-defined interface with normal lungs was visualized, the diagnosis of cyst(s) was made. Septal thickening was considered to be present when there was a prominent thin linear opacity along the interlobular septum at right angles to and in contact with the pleural surfaces. The diagnosis of fibrosis was made when there were reticular opacities and honeycombing (i.e., closely approximated ring shadows typically 3–10 mm in diameter with walls 1–3 mm in thickness, resembling a honeycomb).

#### 2.6.2. Pleural Evaluation on Thoracic MDCT Study

The reviewers evaluated the pleura on the included thoracic MDCT studies for the presence of three main potential abnormalities: (1) pleural thickening; (2) pleural effusion; and (3) pneumothorax [24,25]. The diagnosis of pleural thickening was made when there was an abnormally increased regular or irregular thickness of the lining of the pleura. Pleural effusion was considered to be present when there was fluid within the pleural space. When a pleural effusion was observed, the location (right, left, and bilateral) and size (small (<1/3 of the height of the hemithorax), medium (between 1/3 and 2/3 of the height of the hemithorax), and large (>2/3 of the height of the hemithorax) were also evaluated. The diagnosis of pneumothorax was made when air was present in the pleural space. When pneumothorax was present, the location (right, left or bilateral) and the size (small (<1/3 of the height of the hemithorax), medium (between 1/3 and 2/3 of the height of the hemithorax) or large (>2/3 of the height of the hemithorax) were also evaluated.

In addition, if any additional thoracic MDCT abnormalities besides the previously described lung and pleural abnormality categories on thoracic MDCT studies were observed during evaluation, the reviewers were also instructed to record them.

### 2.7. Statistical Analysis

Continuous variables, such as age, were summarized via means, standard deviations, and ranges. For each type of abnormality, Group 1 (PVS with aspiration) and Group 2 (PVS without aspiration) were compared with respect to the proportion of abnormalities using Fisher exact tests. For the purposes of calculation, extremely sparse tables (no abnormalities in both groups) were augmented by cell counts of 1. The probability of consolidation correctly classifying a pair of PVS patients with and without aspiration was obtained using the c-index of a logistic model in which consolidation was used to predict the presence or absence of aspiration. Interobserver agreement between the two independent reviewers regarding thoracic MDCT findings was measured by the proportion of agreement and the Kappa statistic. As a guide for interpreting magnitudes for the proportion of agreement, we defined low, moderate, and high agreement via the ranges 0.5–0.75, 0.75–0.9, and 0.9–1, respectively. For Kappa these translate to 0–0.5, 0.5–0.8, and 0.8–1, respectively. Fisher exact tests and Kappa estimates were performed using SAS/STAT^®^ 14.1 using the frequency analysis procedure (PROC FREQ), while the LOGISTIC procedure was used to fit the logistic model [26].

## 3. Results

### 3.1. Patient Information

A total of 64 pediatric patients (36 males (56.3%) and 43 females (43.7%); mean age, 1.7 years; range, 1 day–17 years) met Group 1 or Group 2 criteria. Among these 64 patients, 19 patients (29.7%) belonged to Group 1 and the remaining 45 patients (70.3%) belonged to Group 2. There were 19 thoracic MDCT studies from 19 individual pediatric patients with PVS and aspiration (Group 1), and there were 45 thoracic MDCT studies from 45 individual pediatric patients with PVS without aspiration (Group 2).

Group 1 (PVS with aspiration) consisted of 19 pediatric patients (29.7%). There were 11 (57.0%) male and 8 (42.1%) female patients (mean age: 2.1 years; SD: 4.2 years; range: 1 day to 17 years). All patients included in this group had diagnoses of both PVS and aspiration. Presenting clinical signs and symptoms in these 19 pediatric patients in Group 1 included: bradycardia (*n* = 14; 73.7%), hypoxemia (*n* = 13; 68.4%), failure to thrive (*n* = 13; 68.4%), fever (*n* = 12; 63.2%), shortness of breath (*n* = 12; 63.4%), and pulmonary hypertension (*n* = 11; 57.9%).

Group 2 (PVS without aspiration) consisted of 45 consecutive pediatric patients (70.3%). There were 25 (55.6%) male and 20 (44.4%) female patients (mean age: 1.6 years; SD: 2.1 years; range: 3 days to 7 years). All patients included in this group had PVS but were not diagnosed with aspiration or any other lung disorders. Presenting clinical signs and symptoms in these 45 pediatric patients included: pulmonary hypertension (*n* = 31; 68.9%), fever (*n* = 30; 66.7%), failure to thrive (*n* = 28; 62.2%), bradycardia (*n* = 26; 57.8%), shortness of breath (*n* = 23; 51.1%), and hypoxemia (*n* = 16; 35.6%).

Extrapulmonary and 3D MDCT imaging, pathological, and clinical findings for a subset of patients in Group 1 and Group 2 were previously reported [12,17,18,21,22,23,27].

### 3.2. Thoracic MDCT Findings

Table 1 shows the summary of lung and pleural findings on thoracic MDCT studies in Group 1 (PVS and aspiration) and Group 2 (PVS without aspiration).

#### 3.2.1. Lung Findings on Thoracic MDCT Study

**Figure 1:**
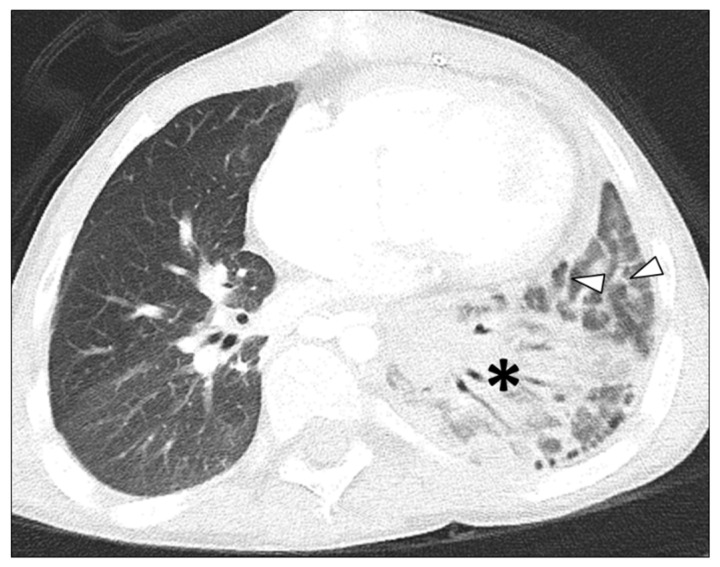
7-year-old male with left sided pulmonary vein stenosis and a history of aspiration. Axial lung window CT image demonstrates consolidation (asterisk) in the left lower lobe and septal thickening (arrowheads).

**Figure 2:**
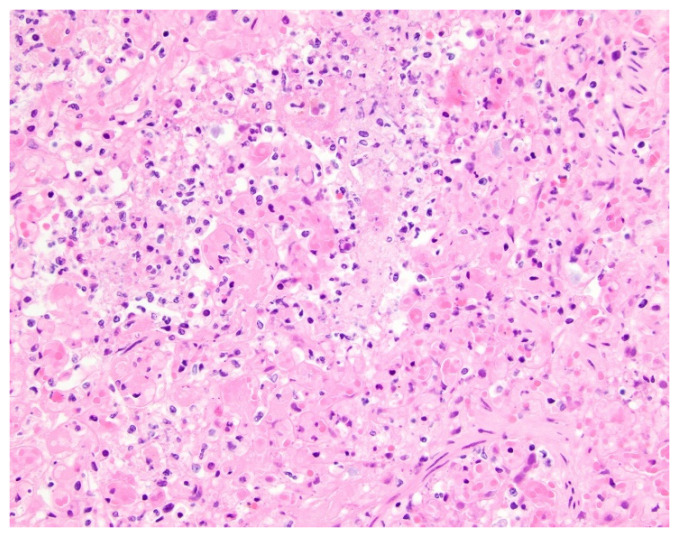
Histologic findings of aspiration in a 10-month-old female with pulmonary vein stenosis. At autopsy, the lungs showed features of pulmonary vein stenosis, and there also was an acute exudative and necrotizing pneumonia. What is pictured is acute exudative pneumonia, with airspaces filled by fibrin, neutrophils, and cellular debris (hematoxylin and eosin stain; original magnification, 400×).

In Group 1 (PVS and aspiration), among 19 thoracic MDCT studies, three lung abnormalities were observed, including GGO in 17 thoracic MDCT studies (89.5%), consolidation in 16 thoracic MDCT studies (84.5%), and septal thickening in 16 thoracic MDCT studies (84.5%) (Figure 1 and Figure 2).

**Figure 3:**
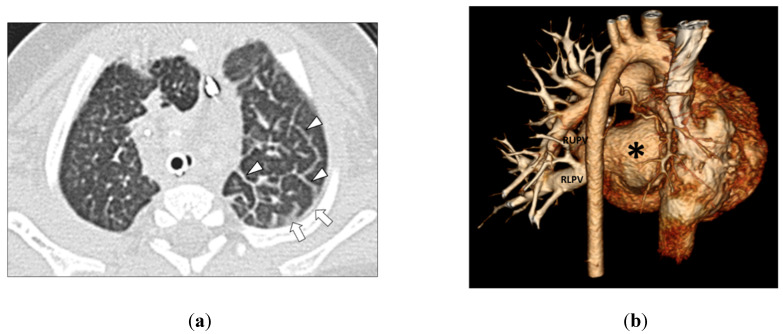
(**a**) 3-month-old girl with left-sided pulmonary vein stenosis but no history of aspiration. Axial lung window CT image shows left-sided septal thickening (arrowheads) and pleural thickening (arrows) in the left hemithorax. (**b**) 3-month-old girl with absent left pulmonary veins due to complete obstruction from severe pulmonary vein stenosis but no history of aspiration. The posterior view of the three-dimensional volume-rendered CT image of the vascular and heart strictures shows absent left pulmonary veins (asterisk). Normal and patent right upper pulmonary vein (RUPV) and right lower pulmonary vein (RLPV) are seen.

**Figure 4:**
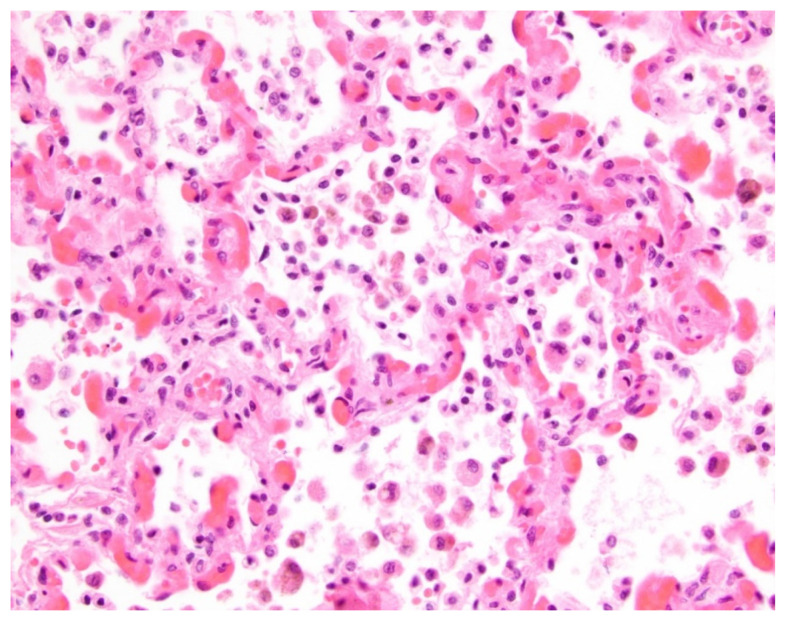
Histologic findings in a 2-year-old male with pulmonary vein stenosis and no clinical history of aspiration. At autopsy, the lungs showed features of pulmonary vein stenosis, including frequent intra-alveolar macrophages, some containing brown hemosiderin pigment. There are also distended alveolar septal capillaries (hematoxylin and eosin stain; original magnification, 400×).

In Group 2 (PVS without aspiration), among 45 MDCT studies, four lung abnormalities were observed including GGO in 37 thoracic MDCT studies (82.2%), septal thickening in 36 thoracic MDCT studies (80.0%), consolidation in 3 thoracic MDCT studies (6.7%), and cyst in 1 thoracic MDCT study (2.2%) (Figure 3 and Figure 4).

The presence of consolidation was significantly more frequently observed in Group 1 (with PVS and aspiration) (84.5% vs. 6.7%, *p* < 0.001). Based on the c-index corresponding to a logistic model in which consolidation was used to predict the presence or absence of aspiration, the probability of consolidation correctly classifying a pair of PVS patients with and without aspiration was 0.89. No other lung abnormalities on thoracic MDCT studies were statistically different between Group 1 and Group 2 (all *p* ≥ 0.525).

#### 3.2.2. Pleural Findings on Thoracic MDCT Study

In Group 1 (PVS and aspiration), among 19 thoracic MDCT studies, pleural thickening was the only pleural abnormality seen in 17 thoracic MDCT studies (89.5%).

In Group 2 (PVS without aspiration), among 45 thoracic MDCT studies, 3 pleural abnormalities were observed including pleural thickening in 37 thoracic MDCT studies (82.2%), pleural effusion in 1 thoracic MDCT study (2.2%), and pneumothorax in 1 thoracic MDCT study (2.2%). Pleural effusions seen in one thoracic MDCT study were bilateral and moderate in size. Pneumothorax seen in one thoracic MDCT study was located in the right side and small in size.

There was no significant difference in the presence of pleural abnormality on thoracic MDCT studies between Group 1 (PVS with aspiration) and Group 2 (PVS without aspiration) (all *p* ≥ 0.710).

### 3.3. Interobserver Agreement

The initial two reviewers were in agreement on all lung and pleural thoracic MDCT findings among all categories, with the exception of 9 occasions (3 occasions in Group 1 and six occasions in Group 2) on the 64 included thoracic MDCT studies.

In Group 1, the three instances of disagreement between the two initial reviewers were related to the presence of septal thickening in two cases and pleural thickening in one case. The third reviewer, the tiebreaker, concluded that septal thickening was present in one case but no septal thickening or pleural thickening was present in the remaining two cases.

In Group 2, the six instances of disagreement between the two initial reviewers were related to the presence of pleural thickening in three cases, septal thickening in two cases, and consolidation in one case. The third reviewer, the tiebreaker, concluded that septal thickening was present in two cases and pleural thickening was present in one case. For the discrepant case related to consolidation, the third reviewer decided that there was atelectasis in the posterior dependent lung rather than consolidation.

There was a high interobserver agreement between the two initial reviewers for detecting lung and pleural abnormalities present on thoracic MDCT studies. The proportion of agreement was 631/640 = 0.99 with 95% CI (confidence interval) (0.979, 0.996), and the Kappa statistic was 0.98 with 95% CI (0.958, 0.992).

## 4. Discussion

The results of our study showed that aspiration is often concomitantly present in pediatric patients with PVS who undergo MDCT, with nearly 30% of children with PVS in our study population carrying a concomitant diagnosis of aspiration. In addition, for the first time, our study found that consolidation is the only pleuropulmonary abnormality on thoracic MDCT that is significantly different in pediatric patients with PVS and aspiration when compared with children with PVS without aspiration. Since consolidation is not a typical radiologic finding of PVS in children, when consolidation is present on thoracic MDCT studies in pediatric patients with PVS, a concomitant diagnosis of aspiration should be considered. The clinical importance of this finding can be described by the c-index corresponding to a logistic model in which consolidation was used to predict the presence or absence of aspiration. This indicated that the probability of consolidation correctly classifying a pair of PVS patients with and without aspiration was 0.89.

We believe that the presence of consolidation, which was the only significantly different pleuropulmonary abnormality on thoracic MDCT in pediatric patients with PVS and aspiration, can be explained by underlying pathophysiology. Aspirated food materials, saliva, and/or oropharyngeal flora introduced into the airways descends through the bronchial tree into lung parenchyma, producing a chemical pneumonitis and/or bacterial pneumonia [28,29,30]. On imaging, such as thoracic MDCT, the resulting acute exudative pneumonia manifests as airspace disease (e.g., consolidation), reflecting the presence of underlying erythrocytes, neutrophils, desquamated epithelial cells, fibrin, and fluid within the alveoli [31,32]. Therefore, the presence of consolidation on thoracic MDCT studies in pediatric patients with PVS should raise suspicion for concomitant aspiration, which has been shown to be associated with poor outcomes in pediatric patients with PVS. However, this is a potentially modifiable risk factor [17]. A recently published study speculated that lung and pleural changes associated with aspiration may directly impact the cells that comprise pulmonary veins’ endothelium, resulting in hyper-proliferation and intraluminal obliteration [17].

Unlike consolidation, GGO is a nonspecific finding in PVS that cannot reliably differentiate between children with PVS with and without aspiration. On thoracic MDCT studies, particularly during the early phase of chemical pneumonitis, aspiration can present as GGO reflecting underlying alveolar inflammation. However, given that GGO was seen in both pediatric PVS patients with and without aspiration (89.5% vs. 82.2%), we believe that the thoracic MDCT finding of GGO cannot be reliably used to differentiate between pediatric PVS patients with or without aspiration. Aspiration-related GGO on thoracic MDCT reflects alveolar inflammation, but the underlying pathophysiology of GGO in children with only PVS (no concomitant aspiration) is different. GGO seen in PVS is felt to mainly reflect alveolar accumulation of transudative fluid from elevated pulmonary venous pressure resulting from obstruction of pulmonary venous return. GGO can also correspond to other pathologic features of PVS, including presence of intra-alveolar hemosiderin-laden macrophages and dilated alveolar septal capillaries characteristic of pulmonary capillary hemangiomatosis-like change [33,34]. High frequency of GGO (90–95%) in pediatric PVS patients on thoracic MDCT has been well documented in several recent scientific investigations and correlates well with findings in this study [21,22,23].

Two additional lung and pleural abnormalities, septal thickening and pleural thickening, were frequently seen in our study population. The underlying pathophysiology of septal and pleural thickening is likely due to elevated pulmonary venous pressure in the interlobular septa and visceral pleural surface, resulting from obstruction of pulmonary venous return [21,22,23]. Because septal thickening (84.5% vs. 80%) and pleural thickening (89.5% vs. 82.2%) were frequently seen in both Group 1 (PVS without aspiration) and Group 2 (PVS with aspiration), we believe that these two characteristic thoracic MDCT findings cannot be reliably used to differentiate between pediatric PVS patients with or without aspiration.

We acknowledge that there are several limitations in this study. First, the patient population is relatively small mainly due to the rarity of PVS in the pediatric population. However, we would like to emphasize that, despite the small study population, the statistically significant finding of consolidation (seen more frequently in pediatric PVS with aspiration) was found in our study, and this new information will be clinically useful for differentiating between pediatric PVS patients with and without aspiration. Secondly, the timing of MDCT studies and the diagnosis of aspiration were not standardized. However, this closely follows the current practice pattern that MDCT study is not routinely performed in all pediatric patients with a new diagnosis of aspiration. Third, there were a few discrepant readings on thoracic MDCT findings between two independent reviewers. However, we emphasize that this is expected since the majority of discrepant readings were related to septal and pleural thickening, which can be subtle findings. In addition, the percentage of discrepant readings between two reviewers was only close to 1% of all readings and interobserver agreement in our study was high. Lastly, pathological confirmation of findings was available in a small number of patients included in this study. However, we would like to emphasize that lung biopsy is an invasive procedure that is not routinely performed in all pediatric patients with PVS. A future study focusing on radiology and pathology correlation of findings in pediatric PVS patients with and without aspiration would be helpful.

In conclusion, our study is the first scientific study to directly compare the lung and pleural abnormalities on thoracic MDCT study in pediatric PVS patients with and without aspiration. We believe that our new finding of the presence of consolidation as a statistically significant differentiating feature, associated with aspiration in pediatric PVS patients, will be valuable for early diagnosis of concomitant aspiration in pediatric patients with PVS in daily clinical practice. Early and accurate diagnosis of this modifiable risk factor (concomitant aspiration) can be potentially critical for improving the outcomes of pediatric patients with PVS because aspiration is associated with poor outcomes in pediatric PVS patients.

## Figures and Tables

**Table 1:** Summary of Lung and Pleural Abnormalities in Children with Pulmonary Vein Stenosis (PVS) with and without Aspiration on Thoracic MDCT Studies.

Types of Lung and Pleural Abnormalities on Thoracic MDCT Studies	Group 1 (PVS with Aspiration) Number (Percentage) of Abnormalities (*n* = 19)	Group 2 (PVS without Aspiration) Number (Percentage) of Abnormalities (*n* = 45)	*p* Value
Lung Abnormalities			
GGO	17/19 (89.5%)	37/45 (82.2%)	0.710
Consolidation	16/19 (84.5%)	3/45 (6.7%)	<0.001
Nodule	0/19 (0%)	0/45 (0%)	0.525
Mass	0/19 (0%)	0/45 (0%)	0.525
Cyst(s)	0/19 (0%)	1/45 (2.2%)	1.000
Septal Thickening	16/19 (84.5%)	36/45 (80%)	1.000
Fibrosis	0/19 (0%)	0/45 (0%)	0.525
**Pleural Abnormalities**			
Pleural Thickening	17/19 (89.5%)	37/45 (82.2%)	0.710
Pleural Effusion	0/19 (0%)	1/45 (2.2%)	1.000
Pneumothorax	0/19 (0%)	1/45 (2.2%)	1.000

MDCT: Multidetector Computed Tomography. PVS: Pulmonary Vein Stenosis. GGO: Ground-glass Opacity.
